# Fluorescence-Based Ratiometric Analysis of Sperm Centrioles (FRAC) Finds Patient Age and Sperm Morphology Are Associated With Centriole Quality

**DOI:** 10.3389/fcell.2021.658891

**Published:** 2021-04-22

**Authors:** Katerina A. Turner, Emily L. Fishman, Mariam Asadullah, Brooke Ott, Patrick Dusza, Tariq A. Shah, Puneet Sindhwani, Nagalakshmi Nadiminty, Emanuela Molinari, Pasquale Patrizio, Barbara S. Saltzman, Tomer Avidor-Reiss

**Affiliations:** ^1^Department of Biological Sciences, College of Natural Sciences and Mathematics, University of Toledo, Toledo, OH, United States; ^2^Department of Urology, College of Medicine and Life Sciences, University of Toledo, Toledo, OH, United States; ^3^Yale Fertility Center, Yale School of Medicine, New Haven, CT, United States; ^4^School of Population Health, College of Health and Human Services, University of Toledo, Toledo, OH, United States

**Keywords:** centriole, infertility, sperm, fluorescence, method, age, teratospermia, miscarriage

## Abstract

A large proportion of infertility and miscarriage causes are unknown. One potential cause is a defective sperm centriole, a subcellular structure essential for sperm motility and embryonic development. Yet, the extent to which centriolar maladies contribute to male infertility is unknown due to the lack of a convenient way to assess centriole quality. We developed a robust, location-based, ratiometric assay to overcome this roadblock, the Fluorescence-based Ratiometric Assessment of Centrioles (FRAC). We performed a case series study with semen samples from 33 patients, separated using differential gradient centrifugation into higher-grade (pellet) and lower-grade (interface) sperm fractions. Using a reference population of higher-grade sperm from infertile men with morphologically standard sperm, we found that 79% of higher-grade sperm of infertile men with substandard sperm morphology have suboptimal centrioles (*P* = 0.0005). Moreover, tubulin labeling of the sperm distal centriole correlates negatively with age (*P* = 0.004, *R* = −0.66). These findings suggest that FRAC is a sensitive method and that patient age and sperm morphology are associated with centriole quality.

## Introduction

Infertility affects 10% of American people, but due to the lack of adequate clinical treatment or access to it, many couples fail to get their desired offspring (Royfman et al., 2020). One reason infertility treatments fail is deficiencies in identifying the underlying causes of male factor reproductive diseases. About 25% of male factor infertility (Pandruvada et al., 2021), 33% of infertility in couples (Smith et al., 2003), 50% of recurrent miscarriages (Garrido-Gimenez and Alijotas-Reig, 2015), and most embryo development defects are unexplained (Reefhuis et al., 2015). Furthermore, intracytoplasmic sperm injection (ICSI) is only successful at leading to clinical pregnancy in 1 out of 2 treated women, and often this requires multiple cycles of treatment (Stern et al., 2010). Therefore, there is a need to identify novel causes of male infertility and develop an assay to diagnose them (Turner et al., 2020).

Centrioles are essential for the sperm’s formation and motility, and the sperm is the sole contributor of centrioles to the zygote (Avidor-Reiss and Fishman, 2019). Recent studies suggest that bovine centrioles play a critical role in coordinating sperm tail and head movement (Khanal et al., 2021). Therefore, sperm centrioles are essential for human fertility (Palermo et al., 1997; Schatten and Sun, 2010; Chemes and Sedo, 2012). The human sperm has two centrioles: a well-established Proximal Centriole (PC) found near the sperm head and a recently discovered atypical Distal Centriole (DC), which is further away from the head and nucleates the axoneme (Fishman et al., 2018). In the testes, the spermatid centrioles undergo a unique “Centriole Remodeling,” which results in sperm centrioles having distinct atypical characteristics (Avidor-Reiss, 2018; Leung et al., 2020). The sperm PC has a canonical structure, but a somewhat atypical composition compared to centrioles in other cells. In contrast, the sperm DC has a dramatically atypical structure and composition compared to centrioles in other cells.

The centrioles’ essential role in sperm morphology and movement pre-fertilization is well demonstrated and widely accepted (Chemes, 2012). The sperm centriole forms the flagellum (i.e., the sperm tail) and may control the tail’s beating shape (Avidor-Reiss et al., 2020). Defects in these functions can result in teratospermia, asthenospermia, or a combination of these phenotypes. Although some of the essential functions of sperm centrioles are known, the canonical PC and atypical DC’s precise role in the mature sperm is still unclear. Furthermore, it may not be possible to circumvent infertility stemming from a defective PC or DC using assisted reproductive technology (ART) such as intracytoplasmic sperm injection (ICSI) with ejaculated or testicular sperm (e.g., testicular sperm extraction, TESE) (Avidor-Reiss et al., 2019). Therefore, patients with defective centrioles may be advised that there is not an effective ART treatment for their condition. Because of the potential impact on infertility treatments, there is a need for more comprehensive studies that directly assess the role of human sperm centrioles (Turner et al., 2020).

Sperm centrioles are essential for embryo development in invertebrates and lower vertebrates (O’Connell et al., 2001; Yabe et al., 2007; Blachon et al., 2014; Khire et al., 2016). However, the essential role of human sperm centrioles in post-fertilization events is still debatable. This controversy is due to two significant issues. (1) Most of the studies that suggest the centrioles are essential after fertilization are descriptive and small scale [Level III, Quality of Evidence, (Force, 1989)]. Available literature includes about a dozen case studies, case-control studies, and a few retrospective studies; no prospective studies were reported (Chemes et al., 1999; Alosilla Fonttis et al., 2002; Nakamura et al., 2002; Rawe et al., 2002; Porcu et al., 2003; Emery et al., 2004; Terada et al., 2009; Moretti et al., 2017, 2019; Sha et al., 2017; Garanina et al., 2019). Most of these reports analyzed less than ten infertile patients. Also, the centriole was only directly examined in a few of these studies, and in many, centriolar defects were inferred from gross cell phenotypes. (2) Sperm centrioles are not essential post-fertilization in mice, and pups can be generated from eggs fertilized by isolated sperm nuclei with no centrioles (Kuretake et al., 1996; Yan et al., 2008). However, the dispensability of sperm centrioles observed in mice does not appear to apply to humans (Avidor-Reiss et al., 2019). Recent studies suggest that bovine centrioles play critical and novel roles in the zygote, and their dysfunction leads to aneuploidy (Cavazza et al., 2020; Schneider et al., 2020). Therefore, it is likely that a fraction of human reproductive diseases may be defined as Sperm Centriole Associated Reproductive Disorders (SCARD).

Assessment of sperm morphology is an essential component of semen analysis and for the diagnosis of male factor infertility (WHO, 2010). Abnormal sperm morphology (teratospermia) is estimated to be present in 7–18% of infertile men and, in the great majority of cases, there is no identifiable cause (Thonneau et al., 1991; Sigman et al., 2009; Owolabi et al., 2013; Peter et al., 2016). Yet, sperm morphology assessment is inherently variable and subjective, and its predictive value for pregnancy success is controversial (Shabtaie et al., 2016). However, pregnancy and live birth rates appear to be lower in some cases of teratospermia (Kruger et al., 1988; Grow et al., 1994; Li et al., 2014; Cito et al., 2020). For example, in oligoasthenoteratospermia (OAT), ICSI treatment results in a lower implantation and gestation rate (Pang et al., 1999; Pfeffer et al., 1999; Rubio et al., 2001). Therefore, to better understand the potential for conception with teratospermic sperm samples, it may help to determine whether such sperm have defects in their internal components, such as DNA, RNA, and centrioles. Consequently, there is a need for a robust method to analyze sperm components, including the centrioles (Turner et al., 2020).

Several assays to study human sperm centrioles are available; however, the associated technology is either laborious, not sufficiently specific to centrioles, or subjective. The structure of sperm centrioles has been assessed by *electron microscopy*, an extremely laborious technique that is inadequate for large-scale studies (Chemes et al., 1987; Moretti et al., 2017). Sperm centriolar protein content has been assessed by *western blot* (Hinduja et al., 2010), which measures total protein and is not specific to the centriole; therefore, it cannot conclusively implicate the centriole in infertility. Sperm centriole function has been assessed by *microinjection* of human sperm into oocytes of bovine, rabbit, or hamster, followed by immunofluorescent staining for aster formation (Nakamura et al., 2001; Terada et al., 2004). These assays are useful for studying abnormal sperm centrioles in some infertility cases (Rawe et al., 2002; Terada, 2004); however, this method creates ethical concerns since it generates an embryo using a human gamete and which is then destroyed. Due to these limitations, sperm centriole defects in infertile men have only been demonstrated in small case studies (Nanassy and Carrell, 2008; Chemes, 2012; Moretti et al., 2017; Sha et al., 2017). Therefore, there is a need for a high-throughput, specific, non-subjective, and quantitative method of assessing human sperm centrioles. Fluorescence microscopy provides a high-throughput technique to predict sperm function based on protein markers, and the centrioles can be specifically analyzed (Petrunkina and Harrison, 2013). However, fluorescence microscopy was used only to identify large defects that include protein mislocalization in small-scale studies (Xu et al., 2008; Sha et al., 2017).

Tubulin and POC1B are structural components of the centriole and axoneme. Tubulin is a heterodimer complex that polymerizes to form the centriole and axoneme wall (Avidor-Reiss and Gopalakrishnan, 2013; Winey and O’Toole, 2014). POC1B is an evolutionarily conserved centriolar protein that forms a luminal scaffold structure in canonical centrioles and novel rod structures in the atypical DC and is essential for centriole stability (Keller et al., 2009; Pearson et al., 2009; Khire et al., 2016; Fishman et al., 2017; Le Guennec et al., 2020). The relationship between the microtubules and POC1B rod structures in the DC is unknown but is likely to have a scaffolding role (Avidor-Reiss and Turner, 2019; Avidor-Reiss et al., 2019).

Advanced paternal age has been consistently implicated in reduced reproductive outcomes and various neurocognitive and developmental conditions in the offspring of humans and other animals (Pizzari et al., 2008; Johnson et al., 2015; Ramasamy et al., 2015; Khandwala et al., 2018). However, the causes for this sperm aging are mostly unknown. One causal factor for sperm aging may be *de novo* mutations in sperm DNA (Kong et al., 2012; Goldmann et al., 2016). However, the contribution of another critical sperm component, the centrioles, to sperm aging is unknown. The centrioles are the only cytoplasmic structure inherited exclusively from the father; they participate in organizing the zygote cytoskeleton and bringing the male and female nuclei together in precise opposition (Sutovsky and Schatten, 2000; Cavazza et al., 2020). As a result, the sperm centrioles are implicated in various reproductive diseases, such as male infertility and early pregnancy failure (Sathananthan, 2009; Chemes and Rawe, 2010; Schatten et al., 2011; Avidor-Reiss et al., 2020). Deterioration of either of the sperm centrioles with patient age may be a novel mechanism for sperm aging.

Here, we developed a quantitative and ratiometric immunofluorescence approach named Fluorescence-based Ratiometric Assessment of Centrioles (FRAC) and performed the most extensive investigation of sperm centriole quality in patients to date using the centriole biomarkers Tubulin and POC1B. We found that infertile men with teratospermia and older men have lower quality centrioles, although the causal relationship between centriolar defects and morphology or age remains unclear. This study opens the door for extensive characterization of the contribution of sperm centrioles to reproductive diseases. Furthermore, rather than providing a binary readout, FRAC quantification of centriole quality provides a range for centrioles quality, which can be combined with other fertility information to specify a more accurate and sensitive fertility prediction. For convenient reading, specific terms (underlined and italicized when they first appear) are described in Box 1.

**BOX 1. Definitions.**

**Centriolar marker or marker** – Either of the antibodies against POC1B and Tubulin.

**Combined mean ratio range** – The combined difference between the patient with highest mean ratio and the patient with lowest mean ratio in the PC, DC, and Ax, and divided by the number of sites.

**Fraction** – The two groups of sperm generated by differential centrifugations: pellet and interface.

**Mean affected individual score (MAIS)** – A score describing the average severity of centriole defects in patient populations with low quality centrioles. The numerator is the number of outlier values added together where >2SD is 1 and >3SD is 2. The denominator is the number of affected individuals.

**Mean ratio** – The mean immunostaining intensity ratio, the mean of the ratios for all sperm in a population from one patient at a location for a marker, usually calculated from 50 to 100 sperm. Each patient had up to 12 mean ratios (PC tubulin, DC tubulin, Ax tubulin, PC POC1B, DC POC1B, and Ax POC1B in the pellet and the interface).

**Mean ratio range** – The difference between the patients with highest mean ratio and the patients with lowest mean ratio from different men of a group (i.e., 0.57–0.25 = 0.32).

**Optimal centrioles** – A patient who had all six mean ratios of the pellet fall within the reference range.

**Optimal reference population** – the 19 men in the reference population that have optimal centrioles.

We studied five populations of sperm: (i) pellet sperm (higher-grade sperm) and (ii) interface sperm (lower-grade sperm) from men with eumorphic sperm; and (iii) pellet sperm and (iv) interface sperm from men with teratospermia; and (iv) pellet sperm from fertile donors.

**Ratio** – The ratios between the staining intensity of one marker in one location (e.g., PC) over the sum of the intensity of the same marker in all other locations in the same individual sperm (i.e., PC/PC + DC + Ax).

**Ratio distribution** – The distribution of single sperm ratios from a single sperm fraction from a single man.

**Reference population** – The pellet sperm from the 22 men with standard sperm morphology; this population includes motility defects and low count.

**Reference range** – The mean ± 2SD of the reference population for a marker at a given location.

**Reference ratio distribution** – The percent distribution of the individual sperm ratios from the 19 men from the reference population that were not outliers.

**Sample (or semen sample)** – the ejaculate or part of it obtained from a sperm donor or patient.

**Severe sub-optimal centrioles** – A patient who had at least one of the six mean ratios fall more than 3 SDs from the reference range mean.

**Sperm location or location** – The three sites in the sperm used when quantifying marker intensity: PC, DC, and Ax.

**Sub-optimal centrioles** – A patient who had at least one of the six mean ratios fall between 2 and 3 SDs from the reference range mean.

**Teratospermia** – Semen condition where sperm have abnormal morphology.

## Materials and Methods

### Overall Project Organization

This project was performed in three consecutive studies over 3 years (November 2016 to September 2019). In each study, sperm centrioles were assessed near the time of collection after the sperm had been separated into a pellet and an interface using differential gradient centrifugation. In study 1 (November 2016 – December 2016), we analyzed the pellet sperm from two proven fertile donors (D1p and D2p) and 12 patients (P1p – P12p) for a total of 14 men obtained from the Yale Fertility Center. In study 2 (May 2018 – September 2018), we received and analyzed the pellet (P13p – P18p) and interface (P13i – P18i) sperm of 7 infertile men from the Urology Clinic at the Regency Medical Center in Toledo. In study 3 (May 2019 – September 2019), we obtained and analyzed the pellet (P19p – P31p) and interface (P19i – P31i) sperm of 12 infertile men from the Urology Clinic at the Regency Medical Center in Toledo. Centrioles were assessed by confocal microscopy using antibody labeling of POC1B and Tubulin. We compared 4 different antibodies against tubulin, including an antibody against acetylated tubulin, and found comparable result in staining of the PC and DC, therefore we choose the sheep antibody because it allowed for more flexibility to stain with antibodies made in rabbit and mouse (Fishman et al., 2018).

### Semen Samples

Semen samples from consenting and de-identified donors or consenting infertile men were produced by masturbation and ejaculated into containers at home or in the privacy of a clinic room. The ejaculates were allowed to liquefy for at least 30 min at 37°C, and basic semen analysis was performed following WHO guidelines, including information on semen volume, sperm count, motility, and morphology (WHO, 2010). This information was used to determine which patients had teratospermia or other sperm infertility phenotypes (Table 1). Samples were frozen in sperm cryopreservation media (TYB, Irvine Scientific) following the manufacturer’s instructions until use, or were used within 5 h of collection (study 2), or were preserved using Sperm CryoProtect (Nidacon, SCP-020) and stored in liquid nitrogen until use (study 3).

**TABLE 1:** Summary of sperm characteristics from semen analysis.

	Infertile Normal morphology (Range, Mean ± SD, N)	Infertile Abnormal morphology (Range, Mean ± SD, N, P)	Donor Normal morphology (Range, Mean ± SD, N, P)
N	22	9	2
Age	19–46, 33.2 ± 6.6, 17	27–46, 35.6 ± 7.9, 9, 0.42	NA
Sperm number	11.7–418, 222 ± 142, 10	82–579, 154 ± 233, 8, 0.46	NA
Sperm concentration	7.8–209, 66.2 ± 61.2, 20	0.3–193, 47.7 ± 73.6, 9, 0.47	40–228, 134 ± 132.9, 2, 0.19
Progressive motility	13–58, 35.9 ± 18.1, 10	0–36, 15.9 ± 13.1, 8, 0.019	NA
Normal morphology	5–16, 8.2 ± 3.6, 9	0–3, 1.2 ± 1.5, 9, 0.0001	NA

**P*-value as determined by *T*-tests comparing Infertile and Donor with Normal morphology. SD, standard deviations; N, number; P, *P*-value; and NA, not available.*

### Differential Gradient Centrifugation, Washing, Attachment, and Fixation

All samples were separated into interface and pellet sperm using differential gradient centrifugation according to the manufacturer’s instructions. The fresh samples were kept at 37°C (study 2). Frozen samples were thawed at 37°C (studies 1 and 3). Then, the samples were layered onto a PureCeption gradient (Origio, ART-2040 and ART-2080) (studies 1 and 2) or a 40/80 PureSperm gradient (Nidacon, PS40-100 and PS80-100) (study 3). The interface was separated from the media and discarded (study 1) or placed in a separate tube (studies 2 and 3) while the pellet remained in the original tube. The interface and pellet sperm were washed using sperm washing media (Origio, ART-1006 (studies 1 and 2) or Nidacon, PSW-100 (study 3). The final pellet of interface sperm and pellet sperm was resuspended in PBS (study 1) or M199 medium (Sigma-Aldrich, M7528) (studies 2 and 3). The total volume of resuspended sperm was 100–600 μL based on the size of the pellet. Approximately 30 μL (study 1), 45 μL (study 2) or 35 μL (study 3) of suspended sperm were pipetted into a single well of a Silicone Isolator (EMS 70339-25) on each of 5 Poly Lysine Slides (VWR 16002-116) (study 1), or at least 3 8mm coverslips for each sample (studies 2 and 3). These slides (study 1) or coverslips (studies 2 and 3) were set to rest for 20 min at room temperature, then the excess liquid was removed, and the silicone isolator was removed (study 1). Either 3.7% formaldehyde (study 2) or methanol (studies 1 and 3) was added as a fixative, and samples were left to rest for 15 min at room temperature (study 2) or 5 min at −20°C (studies 1 and 3). Excess fixative was removed. Slides were then washed three times in PBS before mailing in PBS at room temperature to Toledo, where they were stored at 4°C (study 1) or the coverslips were immediately stained after fixation (studies 2 and 3).

### Sperm Staining

Staining was performed at the University of Toledo. Samples were permeabilized with 0.3% Triton X100 in PBS for 1 h and blocked with 1% BSA in PBS with 0.3% Triton X100 for 2 h (study 1) or 30 min (studies 2 and 3). Primary antibodies diluted in PBS with 1% BSA and 0.3% Triton were applied to the slide (study 1) or coverslip (studies 2 and 3) and incubated overnight at 4°C. Primary antibodies were anti-POC1B [produced in the lab 10537, 1:100, (Fishman et al., 2018)] and sheep anti-Tubulin (Cytoskeleton, Inc.) (1:600 – 2,000). Slides were then washed in PBS with 0.3% Triton X-1000 3 times for 5 min each. Subsequently, slides were incubated with secondary antibodies and 1 μg/100 μL Hoechst. The secondary antibodies were donkey anti-rabbit DyLight 650 (Thermo Fisher Scientific, SA5-1004; 1:300 – 400) or donkey anti-rabbit Alexa 488 (Jackson ImmunoResearch, 711-545-152; 1:300); and donkey anti-Sheep Cy3 (Jackson ImmunoResearch, 713-165-003; 1:1,200 – 2,000) or donkey anti-Sheep Alexa 555 (Thermo Fisher Scientific, A-21436; 1:1,000). Samples were incubated in this solution at room temperature for 4 h (study 1) or greater than 3 h (studies 2 and 3), then washed in PBS with 0.3% Triton X100 3 times for 5 min each, and PBS 3 times for 5 min each. Coverslips were mounted with ProLong gold (Thermo Fischer Scientific, P10144) and allowed to cure for at least 24 h at room temperature in the dark (study 1), or were mounted with Vectashield (Vector Laboratories, H-1200) and sealed with clear nail polish (studies 2 and 3).

### Confocal Microscopy

Slides from all three studies were imaged using a Leica SP8 Confocal microscope in photon counting mode using a 630× magnification, 0.75 zoom, and a format of 4,096 × 4,096 pixels. The scan times was 400 hz with four line averages (study 1) or two frame accumulations and 2 line averages (studies 2 and 3) per scan. We used two sequences. Sequence one activated both a 405 nm laser to collect the DNA staining and the 561 nm laser to collect the tubulin staining; the lasers in this sequence were also used to generate a phase-like picture. Sequence two activated the 633 nm laser (studies 1 and 3) or the 488 nm laser (study 2) to collect the POC1B staining and to generate a phase-like picture. All lasers were kept at less than 5% power. We collected multiple Z sections (10–20) of 0.3 μm thickness from the top of the highest sperm to the bottom of the lowest sperm. We generated a max projection of all the data using the LAX program. Pictures were taken with a gain of 10 and a laser intensity that prevented signal saturation of the centriole markers so we could record both increases and decreases of signal.

**FIGURE 1:**
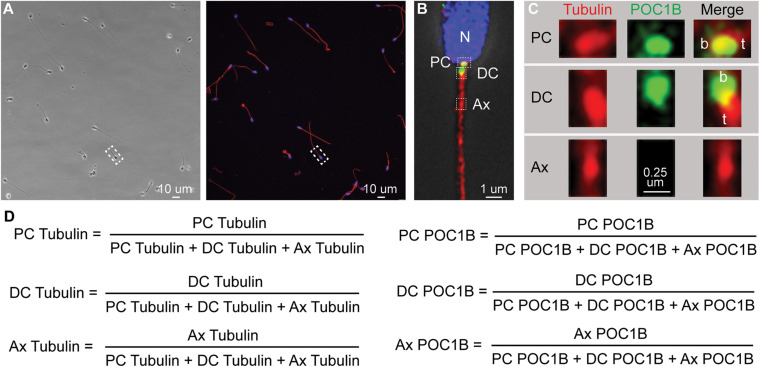
Quantitative imaging of sperm centriole markers. **(A)** An example phase and fluorescent image of a group of pellet spermatozoa. Such a picture ordinarily contains 10–30 spermatozoa. **(B)** Zoom in on one sperm from **(A)** that was deconvolved using HyVolution. Nucleus (N). Three boxes point the approximate location of the PC, DC, and Ax, insets presented in panel **(C)**. **(C)** Zoom in on the Proximal centriole (PC), distal centriole (DC), and axoneme (Ax) of sperm from panel **(B)** labeled with Anti-POC1B and anti-tubulin antibodies. Centriole base (aka proximal end), b; the centriole tip (t) (aka distal end). We consistently find that POC1B labeled more intensely at the proximal end of the tubulin staining. In the PC, this relative position may be because of the presence of microtubular tip extension, known as the centriolar adjunct. In the DC, this relative position may be due to the higher concentration of microtubules at the DC tip. Tubulin staining in the PC and DC is more intense then the nearby axoneme possibly because the PC and DC microtubules were unmasked during centrosome remodeling (Fishman et al., 2018). Because the centrioles are intensely stained with tubulin antibodies, we diluted tubulin antibodies and avoid their exposer to intense laser power that saturates the tubulin labeling. As a result, the axoneme labeling appears punctate. **(D)** The six *mean ratios* by marker and location. The picture is oriented with the PC on the right as describe in Khanal et al. (2021).

Pictures for Figure 1C were deconvoluted with HyVolution II from Leica Microsystems. We used the SVI Huygens Essential program with the standard strategy, no auto-crop was used, and the mounting media refractive index was set for Vectashield (1.457).

### Staining Quantification

The staining intensities for each marker in the acquired images were quantified in the PC, DC, and Ax of each sperm using a 0.75 μm circle (study 1) or a 0.5 μm × 0.75 μm rectangle (studies 2 and 3) (Figure 1). For every sample, every sperm in the view was measured, regardless of an individual’s sperm phenotype (except for one man whose centrioles were unidentifiable).

### Statistical Analysis

Normal distribution was determined after calculating the skewness and kurtosis by the functions SKEW and KURT in Excel. Pearson Correlation R and best fit were calculated by the function CORREL and the automatic linear regression in Excel, respectively. The Person Correlation R^2^ was calculated by RSQ, and the Person Correlation *P*-value was calculated using the function TDIST in Excel. N was calculated using the function COUNT, the T statistic using the equation [R^∗^SQRT(N-2)]/[SQRT(1-R^2)], and the degrees of freedom was calculated by N-2, in Excel. *Z*-tests were calculated on the site https://www.socscistatistics.com/tests/ztest/default2.aspx.

### Ethical Approval

The studies involving human participants were reviewed and approved by the University of Toledo’s IRB Board and Yale’s Human Investigation Committee. The patients/participants provided their written informed consent to participate in this study.

## Results

During our initial characterization of protein levels in the sperm centrioles using quantitative immunofluorescence (photon counting), we found that immunofluorescence intensity values alone are not sufficiently sensitive and reproducible to detect differences in staining across multiple studies. This insensitivity is largely due to the inherent variability of staining and imaging within and between samples and appeared to be unrelated to the biological quality of the sperm. Therefore, we developed the Fluorescence-based Ratio Assessment of Centrioles (FRAC) assay that compares the ratios of immunofluorescence intensity. In this assay, the intensity at different *locations* (PC, DC, or Ax) is compared to the total intensity at those locations for each *marker* (tubulin or POC1B) per sperm, generating 6 ratios (2 markers × 3 locations = 6) (Figure 1). The use of ratios is advantageous because it has the potential to isolate sperm-specific variables from experimental variation (i.e., lot-to-lot variation in antibodies, reagent potency, human error, changes in microscope laser intensity, and other variables that are difficult to control) (Dunn et al., 1994). To overcome the variability of the sperm present in a patient, we calculated the mean of these ratios from multiple sperm. We will refer to the six *mean ratios* by the marker and location [e.g., “POC1B PC” refers to POC1B PC/(POC1B PC + POC1B DC + POC1B Ax) (Figure 1D)].

### Population Definitions

**FIGURE 2:**
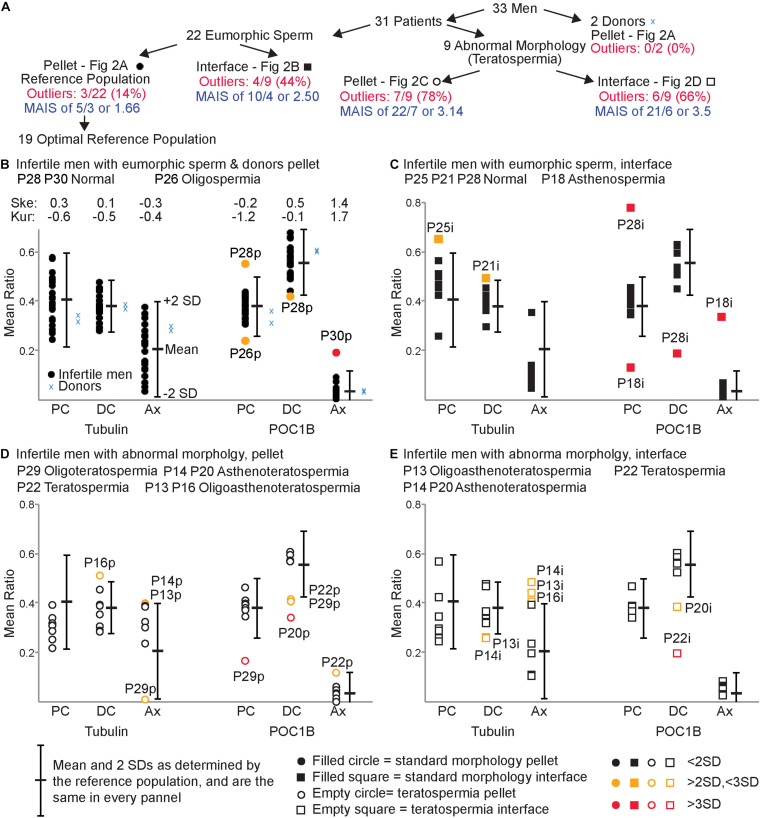
FRAC can identify sperm populations with suboptimal centrioles. **(A)** Breakdown of the 5 populations used. **(B–E)** For each panel, the mean and two standard deviations (SD) of the reference population are indicated to the right of each set of mean ratios. **(B)** Mean ratios of the pellet eumorphic sperm (the reference population) and pellet sperm from donors. The top of the graph indicates the skewness (Ske) and kurtosis (Kur) of the mean ratios of the optimal reference population. **(C)** Mean ratios of the interface eumorphic sperm. **(D)** Mean ratios of the pellet sperm with teratospermia. **(E)** Mean ratios of the interface sperm with teratospermia. Samples that were outside of 2 SD were identified as outliers (colored yellow and labeled). Outliers that were beyond 3 SD are colored red and labeled. The semen analysis results of these outlier patients are identified in the upper left of each graph. MAIS, mean affected individual score.

We analyzed sperm samples from 31 patients and two donors across three studies between 2016 and 2019 (Figure 2A). These 31 patients were all undergoing infertility treatments but had varying diagnoses; their sperm phenotypes were classified as either eumorphic sperm (normal morphology) (*n* = 22) or teratospermia (poor morphology) based on Kruger’s strict criteria (*n* = 9) (see patient details in Supplementary Table 1). Every sample was processed using gradient centrifugation, which resulted in two fractions: a pellet of dense sperm, generally regarded as higher quality, and used for intrauterine insemination, IUI (Karamahmutoglu et al., 2014; Fácio et al., 2016); and an accumulation of lighter sperm at the interface of the gradient phases, which has lower quality sperm (Branigan et al., 1999). The pellet sperm of every sample was collected (*n* = 31), and the interface from studies 2 and 3 were also collected (*n* = 19). This centrifugation produced five groups of sperm, each with hypothetically distinct quality levels: (i) the pellet sperm from men without teratospermia (*n* = 22) (the reference population, Figure 2A); (ii) the interface sperm from men without teratospermia (*n* = 9) (Figure 2B); (iii) the pellet sperm from men with teratospermia (*n* = 8) (Figure 2C); (iv) the interface sperm from the men with teratospermia (*n* = 8) (Figure 2D); and (v) the pellet sperm from fertile men with confirmed fertility (*n* = 2) (Figure 2A). These five groups enabled us to characterize the differences in sperm centriole quality between men with and without teratospermia and examine the efficacy of differential centrifugation in isolating sperm with quality centrioles.

We used pellet sperm of infertile men with eumorphic sperm to overcome the limited availability of fertile donor sperm. Since the population of sperm in an ejaculate is highly heterogeneous (van der Horst and Maree, 2014), we expected that even the most fertile donors will have some sperm with poor centrioles, and the most infertile patients have some sperm with good centrioles. However, we assumed that sperm centriole defects in infertile men with eumorphic sperm would be minimal, and therefore suitable for generating a reference population. The pellet sperm from the infertile men without teratospermia will be referred to as the *Reference Population* (Figure 2B).

### The Reference Population

We analyzed the variation of the mean ratios within the reference population to identify outlier values. To identify the samples with a 95% probability of belonging to the reference population we calculated the mean of the ratios in the reference population ± two standard deviations (SDs), which we called the *Reference Range* (Figurer 2A; Wright and Royston, 1999; Boyd, 2010). Therefore, samples with a mean ratio outside the reference range for *any* of the six mean ratio locations/markers were considered outliers and deemed to have *Sub-Optimal Centrioles*. A sample from the reference population was considered to have *Optimal Centrioles* if all six sperm mean ratios were within the reference range. Nineteen of the twenty-two samples (86%) from the reference population had all six mean ratios fall within the reference range and were considered to have optimal sperm centrioles and we defined as the *Optimal Reference Population* (Figure 2A). The distribution of the optimal reference population is almost normal (Figure 2B). The skewness (the degree of asymmetry of a distribution around its mean) is + /−1 for tubulin of PC, DC, and Ax, as well as for POC1B of PC and DC. Axoneme POC1B skewness was slightly larger, probably because POC1B usually is not found in the axoneme, and the distribution is one-sided. The kurtosis (sharpness of the peak around the mean) is below + /−3 for tubulin of PC, DC, and Ax as well as for POC1B of PC, DC, and Ax.

We determined that three samples from the reference population had sub-optimal sperm centrioles. This result suggested that 14% (3/22) of the reference population samples potentially have sub-optimal centrioles. Two of them, P26p and P28p, had mean ratios between two and three SDs from the mean. The third patient, P30p, had a mean ratio more than three SDs from the mean. P26p, had a semen phenotype of oligospermia (low sperm count), had low PC POC1B; P28p, had a semen phenotype of normospermia (normal semen analysis), had high PC POC1B and low DC POC1B. The third patient, P30p, also had a semen phenotype of normospermia, had high Ax POC1B. This result suggests that there are 4 outlier values to 3 affected individuals, with one that is more than 3 SDs. To evaluate the severity of the low-quality centriole subpopulations we calculated the *Mean Affected Individual Score* (MAIS, The numerator is the number of outlier values added together where >2SD is 1 and >3SD is 2. The denominator is the number of affected individuals). The mean affected individual score is 1.66 (5/3).

Finally, we found that all six mean ratios of the two donor men analyzed fell within 2 SDs of the mean (Figure 2B). This distribution supports the assumption that the optimal reference population resembles fertile men.

### Tubulin Ratios Are Negatively Correlated With Patient Age

**FIGURE 3:**
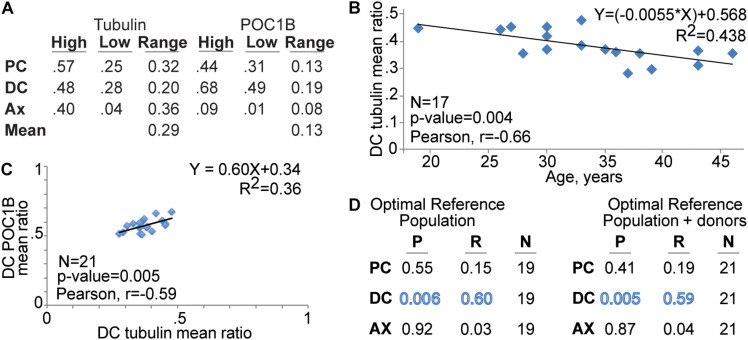
The of DC’s tubulin mean ratio corelate with patient age and DC POC1B. **(A)** Tubulin mean ratio range is wider than POC1B mean ratio range. Table shows the centriole marker mean ratio range of the reference population at the PC, DC, and Ax as well as centriole marker combined mean ratio range in the pellet sperm (Mean). **(B)** FRAC finds that the tubulin level in the DC is reduced with patient age. **(C)** Graph depicting the Correlation of DC POC1B and DC tubulin mean ratios in the Optimal Reference Population. **(D)** Table summarizing the correlation *P* value (P), Pearson *R* value (R), and number of men (N) of Tubulin and POC1B patient mean ratios.

The quality of a marker is defined by several factors, including range narrowness, sensitivity, and specificity (Utt, 2016). Here, we evaluate the narrowness of the range. We determined the *Mean Ratio Ranges* for the optimal reference population for tubulin and POC1B at each location. The mean ratio ranges were determined by the difference between the highest individual patient’s mean ratio and the lowest individual patient’s mean (Figure 3A). The mean ratio ranges for Tubulin were 0.32 in the PC, 0.20 in the DC, and 0.36 in the Ax. The mean ratio ranges for POC1B were 0.13 in the PC, 0.19 in the DC, and 0.08 in the Ax. Therefore, the *Overall Mean Ratio Range*, the mean of the mean ratio ranges, for tubulin was 0.29, and for POC1B was 0.13. This overall difference is statistically significant (*P* = 0.05), suggesting that the tubulin distribution is a more variable than the POC1B distribution.

To gain insight into a potential explanation for the distribution difference, we looked for an age effect on tubulin and POC1B. We analyzed the 17 men in optimal reference population that provided their age. The mean ratio of tubulin in the DC has a statistically significant (*P* = 0.004) moderate negative correlation with age (Pearson correlation coefficient, *R* = −0.66) (Figure 3B). The standard deviation did not change with age (*R* = 0.02, *P* = 0.95). We found no statistically significant and no or weak correlation with age amongst the other five mean ratios (*r* = 0.15–0.24; *P* = 0.34–0.58). The age range in this study was 19–46 (i.e., 27 years difference). The line of best fit between DC tubulin and age (*R*^2^ = 0.438) suggests a yearly reduction of ∼1.2% to a total of ∼24% reduction over 20 years. The age effect on DC tubulin may partially explain the wider distribution of tubulin and suggests that DC tubulin is a sensitive marker to other life circumstances.

In canonical centrioles, POC1B forms a scaffold structure that is attached to the microtubules and controls centriole length and stability (Keller et al., 2005; Blachon et al., 2009; Pearson et al., 2009). Therefore, we examined whether the mean ratios of POC1B and tubulin correlate in the pellet of the optimal reference population with normal sperm morphology. We found that, in the DC, POC1B and Tubulin have a significant and modest positive correlation with each other (*P* = 0.006, *R* = 0.60) (Figure 3C). No significant correlation was observed between POC1B and Tubulin in the PC or Ax (Figure 3D). These data suggest that ∼30% of the DC Tubulin ratio can be predicted by DC POC1B, and vice versa. A similar significant positive correlation was obtained between POC1B and Tubulin when the two donor samples were included with the optimal reference population (*P* = 0.005, *R* = 0.59) (Figure 3D).

### Interface Sperm of Infertile Men With Eumorphic Sperm Have Sub-Optimal Centrioles

As sperm cells differentiate, they lose cytoplasm and become denser; this difference is used to select the better sperm found in the pellet in differential centrifugation (Oshio et al., 1987; Sakkas, 2013). During sperm differentiation, centrioles are also being remodeled (Fishman et al., 2018). Therefore, we hypothesized that the less dense, and thus less mature, sperm found in the interface would have reduced centriole quality. We tested this hypothesis by comparing the mean ratios of the standard sperm morphology (eumorphic) interface population (*N* = 9) to the reference range (Figure 2C and Table 2). We found that 44% of samples from these men had outlier mean ratios (4/9). Two, P21i and P25i, had mean ratios between the two and three SDs away from the mean. P21i, who has a semen phenotype of normospermia, had a high level of DC tubulin. P25i, who has a semen phenotype of normospermia, had a high level of PC tubulin. The other two outlier samples, P18i and P28i, had mean ratios outside three SDs from the mean. P18i, who has a semen phenotype of asthenospermia (reduced sperm motility), had low PC POC1B and high Ax POC1B. P28i, who has a semen phenotype of normospermia, had high PC POC1B and low DC POC1B. This patient (P28) seemed to have severely sub-optimal centrioles in both the interface and pellet; he had four outlier values between both sperm populations (pellet and interface), two of which were outside 3 SDs of the mean. These results suggest that the interface sperm of infertile men with eumorphic sperm tend to contain more sperm with reduced quality centrioles than the sperm in the pellet fraction (4/9 versus 3/22, *P* = 0.06) (Table 2). The 6 outlier values, with 4 that are more than 3 SDs (4^∗^2 + 2^∗^1 = 10), in 4 affected individuals yields a mean affected individual score of 2.5 (10/4), which is more severe than the reference population pellet (1.6).

**TABLE 2:** Summary table comparing the various populations of men examined in the study.

	Infertile Standard Morphology N (patients) (% of population)	Infertile Abnormal Morphology N (patients) (% of population)	*P*-value: Infertile Standard/Infertile Abnormal	Donor Standard Morphology N (% of population)	*P*-value: Infertile/ donor
**Pellet**	***N* = 22**	***N* = 9**		***N* = 2**	

2SD	19 (86%)	2 (22%)	0.0005	2 (100%)	0.6527
>2SD	3 (14%)	7 (78%)	0.0005	0	
>2SD, <3SD	2 (P26, P28) (9%)	4 (P13, P16, P22, P14) (44%)	0.0238	0	
>3SD or no staining	1 (P30) (5%)	3 (P20, P27, P29) (33%)	0.03	0	

**Interface**	***N* = 9**	***N* = 9**		**NA**	

2SD	5 (56%)	3 (33%)	0.3421	NA	
>2SD	4 (44%)	6 (66%)	0.3421	NA	
>2SD, <3SD	2 (P21, P25) (22%)	4 (P13, P14, P16, P20) (44%)	0.31732	NA	
>3SD or no staining	2 (P18, P28) (22%)	2 (P22, P27) (22%)	1	NA	
*P*-value: pellet/interface					
2SD	0.06288	0.59612			
>2SD	0.06288	0.59612			
>2SD, <3SD	0.32218	1			
>3SD or no staining	0.13104	0.59612			

**Total (pellet + interface)**	***N* = 9**	***N* = 9**		**NA**	

2SD	3 (33%)	2 (22%)	0.81	NA	
>2SD	6 (67%)	7 (78%)	0.5961	NA	
>2SD, <3SD	3 (P21, P25, P26) (33%)	3 (P13, P14, P16) (33%)	1	NA	
>3SD or no staining	3 (P18, P28, P30) (33%)	4 (P20, P22, P27, P29) (44%)	p.3421	NA	

**P*-value as determined by *Z*-test. SD, standard deviations; N, number; P, *P*-value; and NA, not available.*

### 78% of Infertile Men With Teratospermia Have Reduced Centriole Quality

Most reports that suggest that human sperm centrioles have a role post-fertilization are from IVF studies with teratospermia patients (reviewed by Avidor-Reiss et al., 2019). Therefore, we hypothesized that a significant portion of infertile men with morphologically abnormal sperm would have suboptimal sperm centrioles. As such, the objective of this analysis was to test if POC1B and tubulin can identify centriolar differences between sperm populations with and without morphological defects.

**FIGURE 4:**
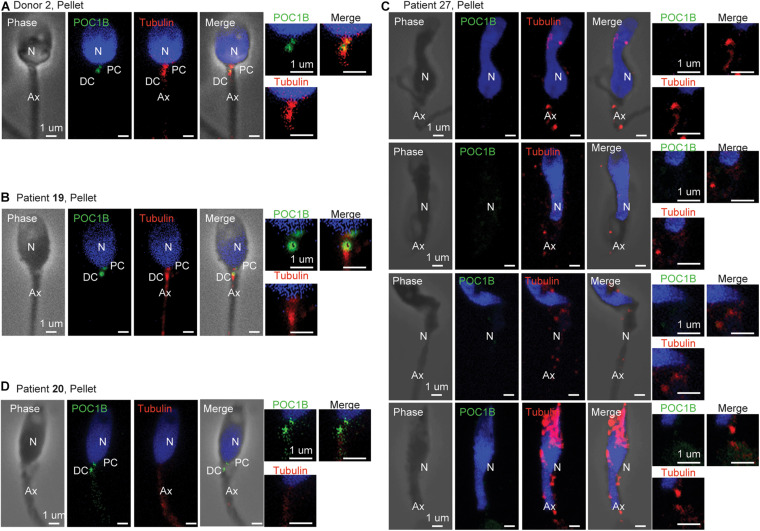
Centriole staining abnormalities. **(A)** Example of standard staining from the pellet sperm of donor 2, who had a standard semen analysis, and whose centriole ratios fell within 2 SDs of the mean for all mean ratios. **(B)** Example of standard staining from the pellet sperm of patient 19, who had a standard semen analysis, and whose centriole ratios fell within 2 SDs of the mean for all mean ratios. **(C)** Example of abnormal staining from the pellet sperm of patient 27, who had a severe oligoasthenoteratospermia phenotype such that the centrioles could not be quantified. **(D)** Example of abnormal staining from the pellet sperm of patient 20, who had an asthenoteratospermia phenotype, and whose centriole ratios fell more than 3 SDs of the mean for distal centriole POC1B. N, nucleus; PC, proximal centriole; DC, distal centriole; Ax, axoneme.

We analyzed the mean ratios of the pellet sperm from nine patients with teratospermia and compared them to the reference range (Figure 2D). For eight of the nine patients, we were able to detect the sperm centrioles using the antibodies, and we analyzed the mean ratio of their pellet sperm (Figure 2D). For one patient, P27, we were not able to detect the PC and DC as discrete entities using either POC1B or tubulin staining in the sperm neck of the pellet or interface populations. Consequently, we were not able to analyze the mean ratios (Figure 4); we concluded that the patient’s sperm had a major centriole defect or that they are missing. Interestingly, this patient was one of three patients with the most severe sperm phenotypes, oligoasthenoteratospermia (OAT) (low sperm count, low motility, and poor morphology), of the samples we studied as determined by traditional semen analysis.

For the remaining eight of the nine teratospermia patients, we found that six had outlier mean ratios of centriole markers (Figure 2D). Four of these patients, P13p, P14p, P16p, and P22p, had mean ratios between two and three SDs from the mean of the reference population. P13p, who has a semen phenotype OAT, had high Ax tubulin. P14p, who has a semen phenotype of asthenoteratospermia, had high Ax tubulin. P16p, who has a semen phenotype of OAT, had high DC tubulin. P22p, who has a semen phenotype of teratospermia, had low DC POC1B and high Ax POC1B. Two of the patients, P20p and P29p, had mean ratios outside three SDs from the mean. P20p, who has a semen phenotype of asthenoteratospermia, had low DC POC1B. P29p, who has a semen phenotype of oligoteratospermia, had low PC and DC POC1B. Interestingly, P13, P27, and P16 all had the same severe sperm phenotype, OAT, but had different severities in their mean ratios, suggesting that semen analysis is insufficient for evaluating sperm quality. These results suggest that the patients with teratospermia have more sub-optimal centrioles than patients without teratospermia (7/9, 78%, versus 3/22, 14%, *P* = 0.0005) (Table 2).

We designated samples with mean ratios outside three SDs or in samples that we were not able to detect the centrioles, P20p, P27p, and P29p, as samples with *severe sub-optimal centrioles*. It also suggests that there is a statistically significant difference in the number of severe centriolar defects (3/9 versus 1/22, *P* = 0.03) (Table 2). This finding supports our hypothesis that sperm with morphological defects are more likely to have lower quality centrioles. This result suggested 14 outlier values (8 + 6) in 7 affected individuals, with 8 that are more than 3 SDs, or a mean affected individual score of 3.14 (22/7), which is more severe than the reference population pellet (1.6) and interphase (2.5).

### Differential Centrifugation Is Insufficient to Differentiate Centriole Quality in Teratospermic Men

We found that differential gradient centrifugation of eumorphic sperm results in a pellet enriched for better quality sperm centrioles. We wondered if differential gradient centrifugation has a similar effect on sperm from patients with teratospermia. Therefore, we analyzed interface sperm from men with teratospermia (Figure 2E). We found that six of the nine interface sperm samples from men with teratospermia had mean ratios outside the reference range. P13i, P14i, P16i, and P20i had mean ratios between the two and three SDs from the mean of the reference population. P13p, who has a semen phenotype of OAT, had low DC tubulin and high Ax tubulin. P14p, who has a semen phenotype of asthenoteratospermia, had low DC tubulin and high Ax tubulin. P16p, who has a semen phenotype of OAT, had high Ax tubulin. P20i, who has a semen phenotype of asthenoteratospermia, had low DC POC1B. One patient, P22i, had a mean ratio more than three SDs away from the mean of the reference population. P22i, who has a semen phenotype of teratospermia, had a low DC POC1B. Again, we were not able to detect the PC and DC as discrete entities in the interface sperm from patient P27, who has a semen phenotype of OAT, using POC1B or tubulin staining. This result suggests that infertile men with teratospermia have a similar incidence of sub-optimal centrioles in the interface and the pellet fractions (6/9 interface versus 7/9 pellet; p is 1) (Table 2). Furthermore, this observation suggests that sperm selection by differential centrifugation is insufficient for increasing the odds of fertilization in cases of sub-optimal centrioles, thus alternative selection techniques should be investigated. Additionally, this result suggests 12 (6 + 6) outlier values in 6 affected individuals, with 7 that are more than 3 SDs, or a mean affected individual score of 3.50 (21/6), which is more severe than the reference population pellet (1.6) or the interface of the patients with eumorphic sperm (2.5).

### The Distribution of a Patient’s Sperm Ratios Provides More Insight on Centriole Quality

While the mean ratios of a patient with optimal centrioles are all within the reference ranges, the individual sperm of that patient may fall outside the reference ranges because sperm populations are heterogeneous. The inverse of this is also true: a patient with sub-optimal centrioles may also have some sperm with optimal centriole ratios. Furthermore, because the sperm is heterogenous, means can oversimplify the data, and specifically ignore bimodal data. Therefore, to gain insight into the heterogeneity of the sperm samples, we analyzed the distribution of POC1B ratios in individual sperm of representative men.

**FIGURE 5:**
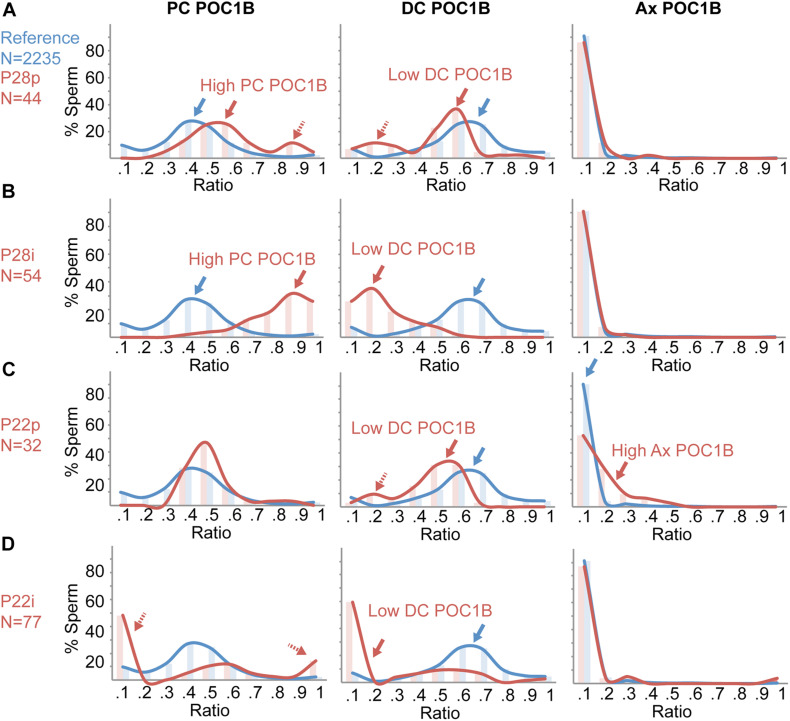
Individual Sperm Ratio Distribution analysis provides additional insight into sperm centriole abnormalities. Histograms of the percent of individual sperm at different ratios of POC1B in the proximal centriole (PC), POC1B distal centriole (DC), and POC1B axoneme (Ax). Reference (blue) is the sperm of the 19 patients within the 22-patient reference population that have optimal centriole quality. **(A)** Ratios of 44 pellet sperm from a patient, P28p (red), that does not have teratospermia but was found to have suboptimal POC1B ratios in the PC and DC. **(B)** Ratios of 54 interface sperm from a patient, P28i (red), that does not have teratospermia but was found to have suboptimal POC1B ratios in the PC and DC. **(C)** Ratios of 32 pellet sperm from a patient, P22p (red), that has teratospermia and was found to have suboptimal POC1B ratios in the DC and Ax. **(D)** Ratios of 77 interface sperm from patient, P22i (red), that has teratospermia and was found to have suboptimal POC1B ratios in the DC. The histogram bins for the ratios on the *x*-axis are 0, –0.1, 0.1 –0.2, 0.2, –0.3, 0.3, –0.4, 0.4, –0.5, 0.5, –0.6, 0.6, –0.7, 0.7, –0.8, 0.8, –0.9, 0.9 – 1.

The individual ratios from all the pellet sperm 19 men in the optimal reference population served as our standard population, which we termed the *Reference Ratio Distribution* (marked with blue in Figure 5). In this reference ratio distribution, POC1B ratios peak at a ratio of 0.3–0.4 for the PC, 0.6–0.7 for the DC, and 0–0.1 for the Ax. The POC1B PC and DC mean ratios had an approximately normal distribution, as the skewness and kurtosis were less than + /−1.0. We then compared it to the staining ratio distributions of a sample of patients that had sub-optimal centrioles, P28p, P28i, P22p, and P22i.

We analyzed the sperm of P28, a patient with eumorphic sperm in the reference population. P28p has sub-optimal higher than the reference ratio distribution peak (0.1) or the pellet sperm (red arrow in Figure 5D, middle panel). Centrioles with two outlier mean ratios (Figure 2B). P28p had high PC POC1B and low DC POC1B (>2SD, <3SD). We looked at the distribution of all P28p’s sperm ratios and found that the PC POC1B ratios peak at 0.5–0.6 (red arrow in Figure 5A, left panel), which is higher than the reference ratio distribution (blue arrow in Figure 5A). Also, P28p has a smaller peak at 0.9, which is not present in the reference ratio distribution (dashed red arrow, Figure 4A, left panel). Interestingly, the peak of DC POC1B ratio mirrors the PC POC1B shift; it was shifted to a lower range of 0.5–0.6 and had an additional peak from 0.1 to 0.3 (red arrows, Figure 4A, middle panel). These two peaks indicate the presence of a subpopulation of sperm with a high PC POC1B ratio, and a low DC POC1B ratio. The correlated shifts in the PC and DC raise the possibility that the increased PC POC1B is at the expense of DC POC1B in P28p. The peak of the Ax POC1B ratio was indistinguishable from the reference ratio distribution (Figure 5A, right panel).

P28i has the same two outlier mean ratios found in the pellet, high PC POC1B and low DC POC1B, but they are more severe (>3SD) than the pellet fraction (Figure 2C). In the interface, the ratio peaks were shifted even further from the reference ratio distribution than the pellet sperm. P28i PC POC1B ratios peaked between 0.9 and 1.0, which is a more exaggerated shift than the pellet population (Figure 5B, left panel). Similarly, P28i DC POC1B ratios shifted to lower than their pellet counterpart to 0–0.2 (Figure 5B, middle panel). Therefore, this analysis indicates that the P28 interface contains more sperm with sub-optimal centrioles. The peak of the Ax POC1B ratio was not unusual when compared to the reference ratio distribution, similar to P28p (Figure 5B, right panel).

Next, we analyzed P22, who has a sperm phenotype of teratospermia. P22 has two mean ratio outliers in the pellet fraction: low DC POC1B and high Ax POC1B (>2SD, <3SD) (Figure 2D). In P22p, the DC POC1B ratio has a peak from 0.5 to 0.6 (red arrow in Figure 5C, middle panel), which is lower than the reference ratio distribution (blue arrow in Figure 5C, middle panel). Additionally, there is a smaller peak at the 0.2 ratio, which is not present in the reference ratio distribution (dashed red arrow, Figure 5C, middle panel). The peak of P22p’s Ax POC1B ratio was slightly higher, near 0.2–0.3, whereas the reference ratio distribution peak was near 0.1 (red arrow in Figure 5B, right panel). These shifted peaks support the possibility that the decreased DC POC1B is due to POC1B leaking from the DC into the Ax in P22p.

P22i has only one outlier mean ratio, low DC POC1B, that it is more severe (>3SD) than the pellet fraction (Figure 2E). As expected, the ratio distribution peak in the interface sperm was higher than the reference ratio distribution peak (0.1) or the pellet sperm (red arrow in Figure 5D, middle panel). Unexpectedly, even though the mean PC POC1B of P22i fell within the ratio range, the ratio distribution analysis shows very few sperm fall into the reference ratio distribution. Instead, there were two opposing peaks at 0–0.1 and 0.9–1 (the peak for the reference ratio distribution is 0.3–0.5). Therefore, the ratio distribution analysis finds that P22i contains sub-optimal centrioles that are missed by the mean ratio analysis.

## Discussion

Fluorescence-based Ratiometric Assessment of Centrioles is the first step toward an assay that can assess sperm centriole quality and reveal their contribution to infertility. We tested two markers: Tubulin and POC1B. However, FRAC can be extended to include other centriole proteins such CETN1, POC5, and CEP135, specific post-translational modifications (e.g., acetylated tubulin), and other subcellular components (e.g., mitochondria, outer dense fibers, manchette, acrosome, nuclear vesicles, etc.). Furthermore, FRAC could be developed for use in other cell types. We demonstrated that an immunofluorescent, localization-based, ratiometric, quantitative assay, such as FRAC, could give reasonably low variability between experiments and enable the identification of outlier samples when comparing distinct sperm populations. We expect that both abnormally low or high levels of any protein at one of the locations (PC, DC, or Ax) may indicate reduced centriole quality, as the deviation of centriole proteins in either direction was associated with sperm dysfunction (Sha et al., 2017; Garanina et al., 2019).

Tubulin and POC1B are components of distinct centriolar substructures. Tubulin is the structural protein of the microtubule (Downing and Nogales, 1998); POC1B is a structural protein of the centriole lumen (Pearson et al., 2009; Khire et al., 2016; Jo et al., 2019; Atorino et al., 2020; Le Guennec et al., 2020). Therefore, we expect that they report on distinct properties of the sperm centrioles. However, whether these proteins or other centriolar proteins are essential for sperm centriole quality is currently unknown. Future elucidation of essential functions will allow for the development of a minimal set of markers that report on distinct sperm centriole functions and could be used to provide a comprehensive sperm quality report.

Here, we used FRAC with sperm from infertile men, but we expect that the reference range obtained from infertile men with eumorphic sperm would resemble those obtained from fertile men. Since sperm centriole defects are likely to affect only a small portion of infertility cases and the sperm from the two fertile donors falls within the reference range it is likely that other fertile men would also fall within the reference range. However, this expectation needs a more formal demonstration with a larger sample size. Also, at this stage of the investigation, it is not clear if the identified outlier ratios have clinical implications. This clarification can be done in the future by correlating centriole quality with infertility treatment outcomes.

Fluorescence-based Ratiometric Assessment of Centrioles is faster than other techniques currently used to assess sperm centrioles. However, it will need further automation and streamlining to be routinely used in a clinical setting. One way would be to use automated slide immunostaining. Alternatively, image-based flow cytometry could also be used to increase the automation of FRAC. Automation of quantification would be possible by writing an algorithm that scans the major features of the sperm, identifies the centrioles, reads the fluorescence intensity, and outputs mean ratios.

One potential concern regarding FRAC is that it may not identify sperm with an overall reduction or increase in centriole staining since it is based on the relative amount of protein levels. We think this only a minor concern because the two centrioles and the axoneme are different structures with different levels of the same proteins. Therefore, we expect that significant changes in protein expression would result in uneven changes to the various structures. This argument is theoretical and will need further validation once hypomorphic mutations in the studied proteins are identified.

Fluorescence-based Ratiometric Assessment of Centrioles has the potential to become a useful tool in the clinical evaluation of semen quality. Applying the findings of this study to the sperm of infertile men in general suggests that a considerable fraction of infertile men have lower quality centrioles. Therefore, large-scale studies of sperm centrioles from fertile and infertile men are needed to determine the clinical cut-off values that are associated with sperm centriole-associated reproductive disease (SCARD).

Aging is an important factor in fertility, especially because the average age of paternity is rising in many countries (Bray et al., 2006). Older men have poorer quality semen, and an increased incidence of health problems in their offspring (Mazur and Lipshultz, 2018). However, very little is known about age related changes to the centrioles, which are usually studied in the context of cancer (Tkemaladze and Chichinadze, 2005; Wu et al., 2020). Due to a lack of a convenient way to study sperm centrioles they have only rarely been studied in the context of aging; therefore, little is known about the effect of age on them. Centriole deterioration with age was observed in fruit flies with centrosome misorientation during sperm stem cell division or centrosome amplification in midgut stem cells (Cheng et al., 2008; Park et al., 2014). Also, the centrosomes’ position changes with age in endothelial cells of the rabbit aorta (Kiosses and Kalnins, 1993). In light of the rarity of studies on age-related effects on centrioles in general, and on sperm centrioles specifically, our finding that the DC changes with age signifies the importance of studying the impact of age on sperm centrioles.

In summary, we have developed FRAC, a new assay based on image quantification, localization, and ratio calculation, that enables the robust assessment of spermatozoa centrioles. Comparing the mean ratio of centriole and axoneme staining in washed, ejaculated spermatozoa could distinguish between men with just teratospermia, from men with teratospermia and sub-optimal centrioles. The mean ratio of spermatozoa in the ejaculate could be used to stratify whether infertile couples are recommended to use sperm donation rather than ICSI; additionally, women with a lower ovarian reserve may opt to store their eggs until the centriole quality of their male partner increases.

## Data Availability Statement

The raw data supporting the conclusions of this article will be made available by the authors, without undue reservation.

## Ethics Statement

The studies involving human participants were reviewed and approved by the University of Toledo’s IRB Board and Yale’s Human Investigation Committee. The patients/participants provided their written informed consent to participate in this study.

## Author Contributions

TA-R conceived the manuscript and wrote it. KT wrote the manuscript, produced the figures, and performed the statistical analysis with the consultation of statistician. BS, EF, MA, and BO collected the data. PD improved staining protocol. EM, PP, TS, and PS collected sperm samples, contributed essential expert information, and helped with the comments for the draft manuscript. NN coordinated IRB activity and sample collection procedures. All authors contributed to the article and approved the submitted version.

## Conflict of Interest

Provisional patent application number 63/117,056 has been filed. The authors declare that the research was conducted in the absence of any commercial or financial relationships that could be construed as a potential conflict of interest.

## Supplementary Material

Supplementary Table 1 Patient information. Excel file containing fertility and semen analysis information of all patients.
